# Thyroidea ima artery multiple branching pattern over the trachea

**DOI:** 10.1007/s00276-023-03156-1

**Published:** 2023-04-27

**Authors:** T. Totlis, K. Natsis, V. Achlatis, T. Pettas, M. Piagkou

**Affiliations:** 1 Department of Anatomy and Surgical Anatomy, School of Medicine, Faculty of Health Sciences, Aristotle University of Thessaloniki, Thessaloniki, Greece; 2 Department of Anatomy, School of Medicine, Faculty of Health Sciences, National and Kapodistrian University of Athens, Athens, Greece

**Keywords:** Thyroidea ima artery, Variation, Thyroid gland, Branch, Origin

## Abstract

**Purpose:**

The current cadaveric report describes a rare case of a thyroidea ima artery (TIA) with multiple branching pattern over the trachea.

**Methods:**

A cadaver dissection of the neck and thorax region of a formalin-embalmed 90-year-old male cadaver of a body donor took place. The body donation was made after a signed informed consent.

**Results:**

The TIA variant originated from the brachiocephalic artery before its bifurcation into the right common carotid artery (CCA) and right subclavian artery (SCA). TIA further divided into three anterior and two posterior branches, with subsequent multiple division into smaller branches. All branches were located anterior and right side to the trachea. The anterior branches supplied the infrahyoid muscles and the posterior ones supplied the thyroid gland inferior lobes and the inferior parathyroid glands. The TIA coexisted with a brachiocephalico-carotid trunk, derived after the left CCA and brachiocephalic artery fusion.

**Conclusion:**

The presence of multiple arterial branches over the trachea creates a high risk for excessive bleeding during tracheotomy or cricothyroidotomy.

## Introduction

A major part of the arterial supply of the thyroid gland (TG) is given from the inferior thyroid artery (ITA, TG lower part and isthmus), while the 36% comes from the superior thyroid artery (STA, TG upper part) [2]. Occasionally, an accessory small artery when present, supplies the TG, the parathyroid glands, and the thymus gland. This artery was characterized as thyroidea ima artery (TIA) or the lowest thyroid artery or the artery of Neubauer and was firstly described in 1772, as a small and inconsistent artery [1], that courses along the trachea anterior surface [16]. TIA prevalence was estimated in 3.8% [16] with a higher prevalence in cadaveric studies (4.3%) compared to imaging studies (3.3%) [16]. TIA may variably be originated from: the brachiocephalic artery (BCA) (74%), the right common carotid artery (RCCA) (9.6%), the aortic arch (7.7%), the right internal thoracic artery (4.8%), the left common carotid artery (LCCA) (1.9%), the left internal thoracic artery (ITA) (1.9%), the subclavian artery (SCA) and the vertebral artery (VA) [16]. TIA may have a variable course and supply to the TG inferior poles and the isthmus. TIA may also coexist with a brachiocephalico-carotid trunk (BCT) (fusion of the BCA with the LCCA), an aberrant right SCA of retroesophageal course, a variable course of the inferior laryngeal nerve (non-recurrent and recurrent) [8] and the ITA bilateral absence [15]. Knowledge of the existence of TIA variants is vital to avoid intraoperative hemorrhage during tracheotomy or cricothyroidotomy [6, 14]. The current cadaveric report describes in detail a rare case of a TIA multiple branching and its clinical significance based on a detail data literature review.

## Case report

**Fig. 1:**
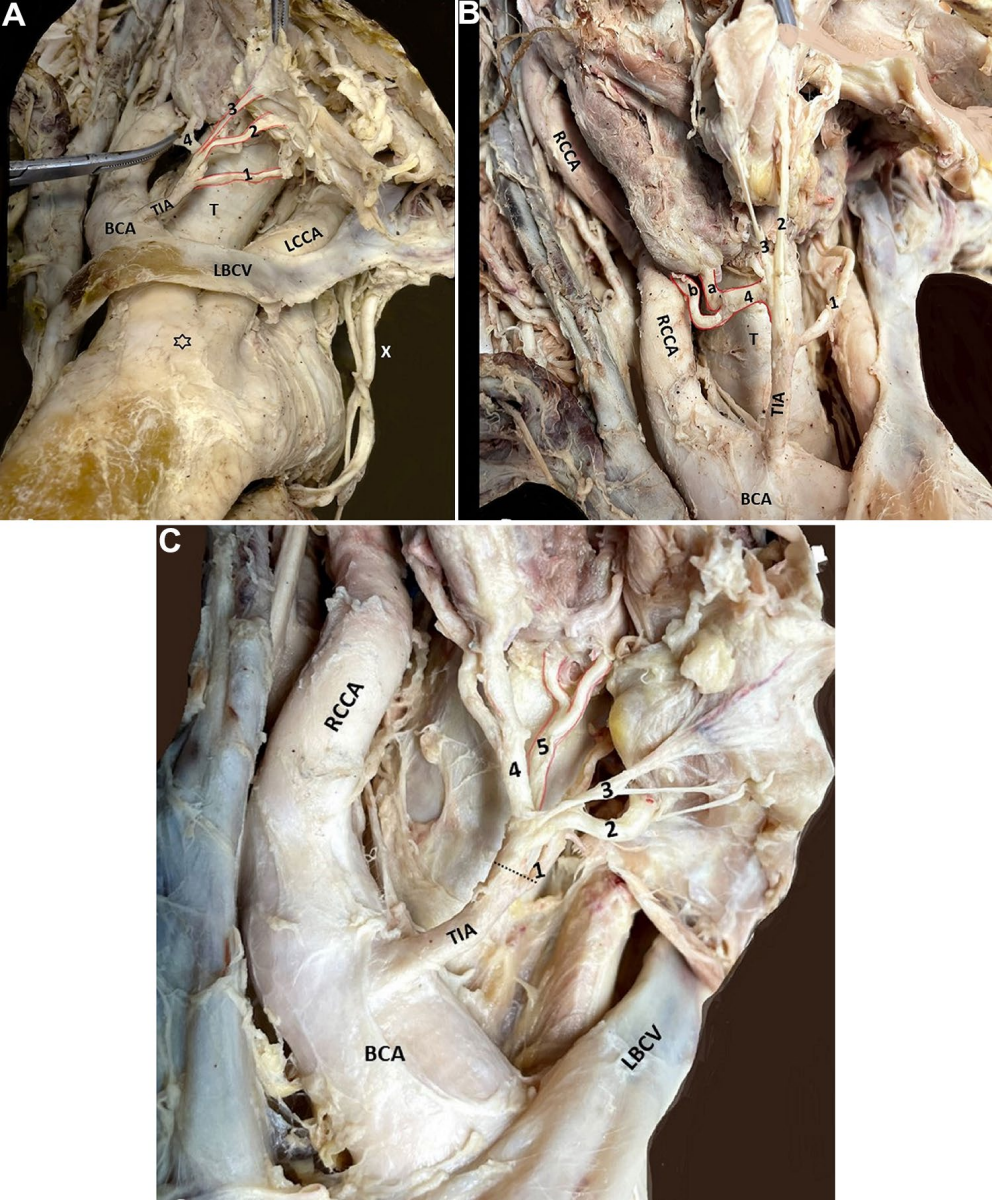
**A** The anterior three-branching pattern from inferior to superior (1,2,3 in red color) of the thyroidea ima artery (TIA), TIA originating from the brachiocephalic artery (BCA), LBCV-left brachiocephalic vein, black asterisk- common origin of the BCA with the left common carotid artery (LCCA) from the aortic arch, X-vagus nerve. **B** TIA lateral 4th branch (4) bifurcation into two branches (a, b in red color), RCCA-right common carotid artery, T-trachea. **C** The thyroidea ima artery (TIA) branching pattern (dotted line-division level) into five branches and further bifurcations. The posterior trunk (5) of the TIA bifurcated into two branches (in red color) directed to the left side, 1- medial 1st branch, 2 and 3- anterior branches, 4- lateral branch division, 5- posterior branch division, *BCA* brachiocephalic artery, *LBCV* left brachiocephalic vein. TIA of parallel course with the LBCV, *RCCA* right common carotid artery (colour figure online)

During a routine dissection of the neck and thorax of a formalin-embalmed 90-year-old male cadaver of a body donor, a rare variant of a TIA was identified. The cadaver was donated to the Anatomy and Surgical Anatomy Department of the Medical School of the Faculty of Health Sciences of the Aristotle University of Thessaloniki. The body donation was made after a signed informed consent. In the right side, an atypical vessel was identified arising from the BCA, before its bifurcation into the RCCA and right SCA. This artery, situated proximal to the left brachiocephalic vein, was identified as the TIA, and further divided into multiple branches (Fig. 1A–C). TIA followed an ascending and recurrent oblique course medially, located anterior and right side to the trachea. The TIA divided into three anterior and two posterior branches, with subsequent multiple division into smaller branches. The anterior branches supplied the infrahyoid muscles (Fig. 1A), and the posterior ones supplied the TG inferior lobes and the inferior parathyroid glands (Fig. 1B). The TIA coexisted with a BCT, derived after the LCCA and BCA fusion (Fig. 1A).

## Discussion

The present report describes an unusual variant and multiple branching pattern of the TIA with clinical impact for tracheotomy and cricothyroidotomy. The TIA, after a recurrent and medial course over the trachea, atypically supplied the infrahyoid muscles, as well as the TG and the parathyroid glands. Banneheka et al. (2010) described a TIA branched off a middle thymothyroid artery (originating from the RCCA anterior surface) and supplied the sternoclavicular joints [3].

### Thyroidea ima artery (TIA) prevalence among studies

Yurasakpong et al. (2022) in their meta-analysis of 36 studies (4335 subjects) estimated the TIA prevalence in 3.8% (with a heterogeneity index 56.2%) [16]. A higher prevalence was detected in Europeans (5.3%) compared to Asians (4%), Americans (2.2%) and Africans (1.6%) [16]. In cadaveric studies (29/36, 2241 cadavers), the prevalence was higher (4.3%, I^2^ = 60%) and lower in imaging studies (3.3%, I^2^ = 48.6%) (6/35, 2014 subjects) [16]. In Natsis et al. (2021) systematic cadaveric review, a TIA was identified with a lower prevalence of 2% [10]. In another imaging study [7], the TIA was identified in 0.16%, confirming the lower prevalence in imaging studies [7]. The TIA may be injured during thyroidectomy or laryngeal transplantation if the surgeon is not aware of it preoperatively [9].

### Developmental anatomy of the TIA

The period between the 3rd and 7th gestational week includes the aortic arch branching pattern formation and the TG synchronous descend [11]. An extensive arterial supply to the TG takes part, and most of the arteries regress, leaving behind STA and ITA. Excessive vascularization may provide pathways for TG morphogenesis and migration and ensures efficient release of thyroid hormones into circulation. The disruption between angiogenesis and TG morphogenesis may be the etiology of TG defects and cardiovascular variants, which may also explain the TIA occurrence [11]. Yurasakpong et al. (2022) highlighted the TIA higher prevalence in fetuses (14.8%) compared to adults (3.3%) (4.5 times more prevalent in fetuses), a finding that justifies the TIA resorption during development [16]. Robinson (1975) identified the TIA premature involution in patients with conotruncal cardiac anomalies, due to hemodynamic alterations [12]. Deficiencies of thymus, and parathyroid glands may be the consequence of vascular deprivation during embryogenesis.

### Thyroidea ima artery (TIA) variable origin

Yurasakpong et al. [16] found the IMA originating from the BCA (in 74%), from the RCCA (in 9.6%), from the aortic arch (in 7.7%), from the right ITA (in 4.8%), from the LCCA (in 1.9%) and from the left ITA (in 1.9%) [16]. In Natsis et al. (2021) cadaveric review, the TIA was also identified to originate from the pericardiophrenic artery, the SCA, the thyrocervical trunk, the inferior thyroid, or the transverse scapular artery [10]. Natsis et al. (2015) in their reports’ review, from 1941 to 2015 [8] recorded the TIA origin from the right SCA in common trunk with the right vertebral artery in 20%. Yohannan et al. [15] reported an unusual origin from the right SCA very close to the right vertebral artery.

### Thyroidea ima artery variable course and supply

Fujimoto et al. (1974**)** recorded a TIA from the BCA anteromedial wall, supplying the anterior and posterior surfaces of the lower isthmus area and the sternothyroid muscles of both sides [5]. The TG isthmus is usually supplied by the ITA or less commonly by the BCA or the aortic arch [4]. Yohannan et al. [15] reported an atypical anterior course of the TIA (between CCA medially and internal jugular vein laterally). The TIA coursed until reaching the TG inferior pole of the right lobe and branched to supply the anteroinferior and posteroinferior aspects of both lobes and isthmus.

### TIA coexistence with other variants

In Natsis et al. (2021) systematic cadaveric review, the TIA was combined with a BCT with a prevalence of 1% [10]. The TIA may also coexist with an aberrant right SCA of retroesophageal course, a non-recurrent laryngeal nerve (in 60%) and a recurrent laryngeal nerve (in 40%) [8]. Yohannan et al. [15] reported the TIA coexistence of unusual origin and course with a bilateral absence of the ITAs. In Natsis et al. (2017) study, a rare combination of the aberrant right SCA with a TIA was detected in 0.4%, where TIA emerged from the RCCA [9].

### Clinical impact

Awareness and preoperative knowledge of TIA is vital for its importance during neck and thoracic surgery. The cricothyrotomy and tracheostomy should be performed with extremely caution, particularly due to the possible existence of TIA and the serious intra-operative bleeding and ischemia, after its injury. Kamparoudi et al. [6] published a case report where existence of TIA during percutaneous dilatational tracheostomy caused excessive bleeding. A patient having a TIA variant with multiple branches in front of the trachea, such as the one presented in the current study, is more prone to those complications. Thus, the TIA is essential to be detected preoperatively, under ultrasound sonography. Ultrasound-guided percutaneous tracheostomy is a safe procedure and reduces the risk of bleeding [13]. Whenever the TIA is identified, it should be preserved because in many individuals, it replaces the ITA by supplying the TG and PGs [4]. Since TIA mostly originates from the right side, the endotracheal tube should be inserted on the left side of the midline to avoid potential TIA injury. Although TIA presence is asymptomatic, its identification is of immense significance, since missed adenomas or hyperplastic gland lesions may be revealed, during parathyroid arteriography, if TIA is present [9].

## Conclusion

In the current study, a TIA with multiple branches running over the trachea was described. The TIA supplied the thyroid, parathyroid and atypical supplied the infrahyoid muscles. The presence of multiple arterial branches over the trachea creates a high risk for excessive bleeding during tracheotomy or cricothyroidotomy.

## Data Availability

Not applicable.
